# Hereditary haemorrhagic telangiectasia and pregnancy: a review of the literature

**DOI:** 10.1186/s13023-019-1286-z

**Published:** 2020-01-07

**Authors:** Olivier Dupuis, Laura Delagrange, Sophie Dupuis-Girod

**Affiliations:** 1 Hospices Civils de Lyon, Service de Gynécologie-Obstétrique, Hôpital Lyon-Sud, Pierre-Bénite, France; 2 Université de Lyon, Faculté de médecine, Lyon, France; 3 Hospices Civils de Lyon, Centre de Référence pour la maladie de Rendu-Osler, Hôpital Femme-Mère-Enfants, Bron, France; 4 Université Grenoble Alpes, Inserm, CEA, BIG-Biologie du Cancer et de l’Infection, Grenoble, France

**Keywords:** Hereditary haemorrhagic telangiectasia, Pregnancy, Arteriovenous malformation, Haemothorax, Haemoptysis, Stroke

## Abstract

**Background:**

Hereditary haemorrhagic telangiectasia (HHT) is a dominantly inherited genetic vascular disorder that has prevalence of 1:5000 to 1:8000, and which is characterised by recurrent epistaxis, cutaneous telangiectasia, and arteriovenous malformations (AVMs) that affect many organs including the lungs, gastrointestinal tract, liver, and central nervous system.

The aim here was to carry out a review of the literature on HHT complications during pregnancy in order to guide management decisions.

**Main body:**

A literature review was carried out to analyse all publications on complications that occurred during pregnancy in women with HHT. The PubMed/Medline and Scopus databases were searched. The complications observed in HHT women during pregnancy were then described.

The authors identified 5 case series and 31 case reports that describe the evolution of 1577 pregnancies in 630 women with HHT. The overall maternal death rate described in the case series was estimated at 1.0% of pregnancies in the case series and 2 maternal deaths occurred in 31 pregnancy case reports. Severe maternal complications occurred in 2.7 to 6.8% of pregnancies in the case series. Severe complications occurred mostly in the second and third trimester in non-diagnosed and non-screened HHT patients. Severe complications were related to visceral involvement. The most frequent complications were related to pulmonary arteriovenous malformations (PAVMs) (haemothorax (*n* = 10), haemoptysis (*n* = 4), and severe hypoxaemia (*n* = 3)). Neurological complications were related to PAVMs in one case (right to left shunt) and to cerebral arteriovenous malformations (CAVM) and intracranial haemorrhage in 2 cases. Complications were related to hepatic arteriovenous malformations (HAVMs) in 8 cases (acutely decompensated heart failure due to hepatic involvement (*n* = 1), dyspnoea related to heart failure (*n* = 5), and hepatobiliary necrosis (*n* = 2)).

**Conclusion:**

Based on the literature review, most pregnancies in HHT women occur normally. However, these pregnancies should be considered high-risk, given the potential life-threatening events related to AVM rupture. Furthermore, there is currently no international consensus regarding the medical follow-up of pregnancy in women with HHT and the aim here was to carry out a review of the literature in order to guide screening and management decisions for this rare disease.

## Introduction

Hereditary haemorrhagic telangiectasia (HHT) is a dominantly inherited genetic vascular disorder characterised by recurrent epistaxis, cutaneous telangiectasia, and visceral arteriovenous malformations [1]. Clinical diagnosis is based on the Curaçao Criteria defined in 2000 by the Scientific Advisory Board of the HHT Foundation International Inc.; these consist of the following 4 signs: 1. epistaxis that occurs spontaneously on more than 1 occasion; 2. telangiectasias at characteristic sites including the nose, fingers, and oral cavity; 3. visceral lesions such as pulmonary, hepatic, or cerebral arteriovenous malformations (AVMs); 4. a family history of HHT (first-degree relative diagnosed with HHT via the same criteria). To be diagnosed with HHT, a patient must meet at least 3 of the 4 criteria [1, 2]. HHT is caused by mutations in *ENG* (encoding endoglin) [3], *ACVRL1* (encoding activin receptor-like kinase 1) [4], or *MADH4* (encoding SMAD4), which are responsible for an imbalanced state between anti- and pro-angiogenic factors, such as vascular endothelial growth factor (VEGF) [5]. More than 90% of all cases of HHT are due to mutations in either *ENG* or *ACVRL1*.

Pregnancy, via the associated hormonal changes, promotes modification of the vascular bed and can have an impact on the disease [6]. This corresponds to systemic vasodilatation and a progressive decrease in peripheral vascular resistance until the middle of the second trimester, before beginning to increase late in the third trimester. There is also an increase in cardiac output; the greatest increase, of up to 45% from baseline, occurs during the first trimester. The increase in cardiac output slows late in the second trimester and drops slightly late in the third trimester [6]. The haemodynamic changes of pregnancy thus exacerbate blood shunting through already abnormal vascular beds [7].

In HHT, complications during pregnancy are rare but can be severe. Screening for pulmonary AVMs is recommended in all cases before pregnancy, as well as pulmonary AVM (PAVM) embolotherapy if possible [8]. However, symptoms may reach clinical thresholds for the first time during pregnancy or after delivery due to PAVMs.

## Search strategy

We performed a literature search using the PubMed/MEDLINE and Scopus databases between March and April 2019 to identify all original articles evaluating or reporting complications during pregnancy in women with HHT. The search strategy was based on a combination of key words and controlled vocabulary for HHT and pregnancy such as “Hereditary Haemorrhagic Telangiectasia” or “Osler-Weber-Rendu disease” or “Osler-Weber-Rendu syndrome” and “pregnancy” or “pregnant woman” or pregnancy outcomes”. No date or language limits were used.

Studies were excluded if they were not in English or French or if they did not present data about HHT and pregnancy. Articles were double screened by two reviewers (SDG & LD) based on title and abstract to determine whether they met the inclusion criteria for a full-text review. Articles without abstracts that appeared potentially relevant based on their title were kept for further consideration. Full text screening was completed by all authors.

Data and collection process: data regarding the number of patients and pregnancies, date of events (in gestational week), clinical presentation and treatments, term of delivery, obstetric events, route of delivery, and maternal and foetal outcome were extracted.

## Results

### Study selection and characteristics

**Fig. 1:**
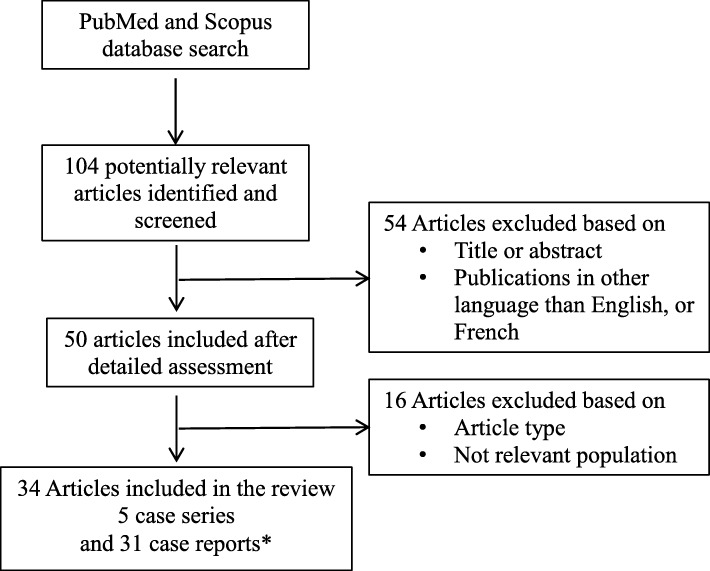
Study selection flow chart for final inclusion in analysis. * 2 case-report in the same article (*n* = 2)

A total of 104 studies were identified after screening the title and abstract, 50 records were retained and underwent a full text examination, and 34 articles were included in the review (5 case series, 31 case reports; Fig. 1), representing a total of 630 women with HHT and 1577 pregnancies. The characteristics of the 5 case series are summarized in Table 1, and those of the 31 case reports in Table 2.

**Table 1:** Characteristics of pregnancies in women with HHT: A summary of 5 case series published between 1967 and 2014

Study (Author, type)	Women (n) / Pregnancies (n)	Live births (n)	Results	Death n (% of pregnancies)	Known diagnosis (%) / rate of patients screened for PAVM / CAVM	Obstetrics events	Route of delivery V D (%) / C S (%)
Goodman et al. 1967 Retrospective	40 / 97 + 80/213 controls	78	no statistically significant difference between the 2 groups (HHT and control)	NA	100 / - / -	Miscarriage 14.4 vs 10% Abnormal outcome 50 vs 42% Prematurity 9.4 vs 10.3% Stillbirth 3.1 vs 2.6%	NA
Shovlin et al. 1995 Retrospective	47/161	NA	11 severe events (6.8% of pregnancies) PAVM shunt deterioration *n* = 6 Cerebro vascular accident *n* = 3 Fatal Pulmonary hemorrhage *n* = 2 10/11 events in the « PAVM + » group	3 (1.9)	100 / - / -	No Significant difference in the Miscarriage rate between the « PAVM + » and « PAVM – » group	NA
Shovlin et al. 2008 Prospective+retrospective	199/484	NA	13 severe events (2.7% of pregnancies) Myocardial infarction *n* = 1 (died) Cerebro vascular accident *n* = 6 (2 died / 2 ischaemic, 3 haemorrhagic, 1 NA) Pulmonary hemorrhage *n* = 6 (2 died)	5 (1)	26 / - / -		NA
Wain et al. 2012 Retrospective	226/560	457	NA	NA	100 / - / -	T1 Miscarriage 14.3% Preeclampsia 0.6% PPH 6.2% Prematurity 13.8% LBW 10.7%	73.5 / 20.6
De Gussem et al. 2014 Retrospective	87 / 244	185	15 severe events (6.1% of pregnancies) Heart failure *n* = 1 Hemothorax *n* = 4 (4 undiagnosed PAVMS) Hemoptysis *n* = 2 Transient Ischaemic attack *n* = 2 Post partum: Deep veinous thrombosis *n* = 1 Pulmonary embolism *n* = 1 Myocardial ischemia *n* = 2 Intracranial hemorrhage *n* = 1 Hemothorax *n* = 1	NA	100 / 13 / 17	Miscarriage 20% Gestational hypertension 2.7% Preeclampsia 7% Eclampsia 0.5% Diabetes 3.2% PPH 2% Prematurity 12%	70 / 30
Total	599 / 1546	720					

Legends: *VD* Vaginal delivery*, CS* Cesarean section*, PAVM* Pulmonary arteriovenous malformation*, CAVM* Cerebral arteriovenous malformation*, LB* Live birth, *PP* Post partum, *PPH* Post partum hemorrhage, *LBW* Low-birth weight, NA Data Not Available

**Table 2:** Severe complications occurring during pregnancy or post-partum in women with HHT and published as case report (*n* = 31)

Complication	Study	Treatment T: Transfusion S: Surgery E: Embolisation D: Drain	Date of events (wg)	Clinical presentation	Term of delivey (wg)	Maternal outcome	Fœtal outcome
Haemothorax	Texier et al. 2018	T + S/HHT–	26	Chest pain	40	Good	Live infant
	Md Noh et al. 2018	E/HHT–	20	Dyspnea	20	Small bowel active hemorrhage	Foetal death
	Raiya et al. 2017	D + E/HHT–	23	Dyspnea, chest pain	40	Good	Live infant
	Jakobi et al. 2001	S/HHT+	26	Severe hypoxemia	40	Hypoxemia PP embolisation	SGA infant
	Adegboyega et al. 1996	D/HHT–	29	Dyspnea Chest pain	40	Discharged no sequelae	Live infant (CS)
	Freixinet et al. 1995	D + S/HHT?	27	Dyspnea	?	Severe mitral regurgitation	Live infant
	Bevelaqua et al. 1992	D + E/HHT–	26	Dyspnea Chest pain	40	Diagnosed 6 wk	Live infant
	Laroche et al. 1992	D + S / HHT +	29	Dyspnea Chest pain	37	Post embolotherapy	Live infant
	Gammon et al. 1990	D + E / HHT -	24	Dyspnea Chest pain	30	Heart failure resolved	Live infant
	Waring et al. 1990	D + E/HHT +	26	Dyspnea	32	Heart failure resolved	Live infant
Hemoptysis	Banerjee et al. 2018		34	Hemoptysis	–	Good	–
	Tandon et al. 2017	reE of PAVM/HHT+	32	Unconscious and hypoxia	37	Good	Live infant (VD)
	Yaniv-Salem et al. 2017	reE of PAVM/HHT+	35	Massive hemoptysis	37	Good	Live infant (VD)
	Wispelaere et al. 1996	−/HHT+	10	Hemoptysis, transient loss of consciousness and tachycardia	Therapeutic abortion	Well, required resection of PAVM	–
Severe Hypoxemia	Worda et al. 2007	−/HHT+	12	Dyspnea, cyanosis	32	Clinically improved	Live infant (CS)
	Jakobi et al. 2001	−/HHT+	25	Hypoxemia IUGR	25	–	Foetal death
	Swinburne et al. 1986	−/HHT+	35	Dyspnea, cyanosis	35	Post partum PAVM surgery Active limited live	Live infant (CS)
Cerebral Ischemic stroke	Swietlik et al. 2008	Craniotom/HHT+	35	Headache and dyspnea Brain abscess + PAVM	35	Post partum Hemothorax requiring re Embolisation	Live infant (CS)
Intracranial hemorrhage	Gillard et al. 1996	CAVM surgery/HHT–	21	Right hemiplegia, aphasia	38.5	Post partum epilepsy, Right hemiparesis	Live infant (CS)
	Neau et al. 1988	Brain surgery/HHT–	30	Right hemiplegia with aphasia violent headache and vomiting	30	Fatal (multiple CAVM, 5 hematomas)	Foetal death (VD)
Pulmonary edema	Euser et al. 2012	HHT+	33	Pre-eclampsia, extensive edema in her legs and face	34	Good	Live infant (CS)
High output heart failure all related to liver AVM	Berthelot et al. 2015	Diuretics/HHT–	25	Dyspnea	33	At day 16 post partum complete regression of congestive signs	Live infant (CS)
	Lai et al. 2010	?/HHT+	36	Rest Dyspnea	36	Good	Live infant (CS)
	Goussous et al. 2009	?/HHT-	29	Right sided heart failure and preterm labor		On post partum day 2: dyspnea and lower extremity edema	? (CS)
	Livneh et al. 1988	Diuretics/HHT+	26	Weakness, dyspnea	35 (?)	At 4 months Post partum Good	Live infant
	Livneh et al. 1988	Diuretics/HHT–	26	Dyspnea	40	At 4 months Post partum no more heart failure	Live infant
Hepatobiliary necrosis	McInroy et al. 1998	Post partum Liver transplantation HHT+	30	Abdominal pain + fever	30	Biliary necrosis, liver transplant postpartum	Live infant
	Bauer et al. 1995	Liver transplant HHT–	–	Abdominal pain dyspnea, Melaena	–	Liver transplant	–
Gastrointestinal bleeding	Hillert et al. 2001	Liver transplantation/HHT+	27	Diffuse abdominal pain cholangitis	29	Good	Live infant (CS)
Branch retinal artery occlusion	Askim et al. 2017	Subcutaneous Heparin + PAVM embolization/HHT–	12	Sudden painless scotoma in left eye	40	Good	Live infant (VD)
Massive intraperitoneal haemorrhage	Sivarani et al. 2010	HHT+	36	High-output cardiac failure	36	Fatal (on post-partum: massive haemorrhage in the intra-peritoneal cavity with multiple AVMs in the gastrointestinal tract)	Live infant (CS)

Legends: HHT- means that the diagnosis was not done before the complication and HHT+ means that the diagnosis was known before the complication. *CS* Cesarean section, *PAVM* Pulmonary arteriovenous malformation, *SGA* Small for gestational age*, VD* Vaginal delivery

### Case series

The 5 case series corresponded to a total of 1546 pregnancies in 599 HHT-affected women and 720 live births (unknown information in 2 series) [8–12]. The HHT diagnosis was known before the pregnancy in 4 series [8], and all five studies were retrospective although one study [8] had a prospective component. Severe events occurred in 2.7, 6.1 and 6.8% of pregnancies as summarized in Table 1. Two case series reported maternal death during pregnancy [8, 10]; there was a total of 8 deaths in 645 pregnancies in 246 women (1.2% of pregnancies and 3.3% of affected women). PAVM-related complications (hypoxaemia and/or haemorrhage and/or ischaemic stroke) were the most frequent severe event described in HHT women during pregnancy, with a total of 26 events related to PAVM. Furthermore, out of 6 pulmonary haemorrhages published by Shovlin et al. [8], four women had previous PAVM screening and embolisation. Cerebral AVM (CAVM)-related complications (intracranial haemorrhage) were observed in 4 cases and other complications (myocardial infarction, heart failure, deep venous thrombosis) in 7 cases [8, 10, 12] (Table 1).

### Case reports

A total of 31 case reports of patients with HHT and life threatening events occurring during pregnancies were identified from the literature available. Mean gestational time at the presentation of the adverse events was 27 weeks of pregnancy (range: 20–36). Most complications appeared at the end of the second trimester or at the beginning of the third trimester of pregnancy.

Two maternal deaths and 2 foetal deaths occurred in 31 pregnancies in this series of case reports [13, 14]. One was due to successive lobar intracerebral haematomas in a 19-year old at 30 weeks of gestation, and the second was due to a massive haemorrhage in the intra-peritoneal cavity with multiple AVMs in the gastrointestinal tract.

The most frequent complications were related to PAVMs. Ten women had acute PAVM-related haemothorax [15–24] and of them, 3 had been screened for PAVMs before pregnancy or even been aware of their HHT diagnosis [18, 22, 24]. Haemoptysis during pregnancy occurred in 4 HHT cases [25–28]. Two were weak and both women were able to give birth to live infants at 37 weeks [26, 27]; one woman had been diagnosed and treated for PAVMs 5 years previously. Recanalization of the pulmonary AVM sac, necessary partly because of the pregnancy, occurred with subsequent haemoptysis. Despite successful therapeutic re-embolisation of the afferent pulmonary artery, haemoptysis recurred 5 days later [28]. Three women presented with dyspnoea related to severe hypoxaemia [18, 29, 30] and early-onset foetal growth restriction resulted in foetal death at 25 weeks’ gestation in one case [18].

Neurological complications were related to PAVMs in 1 case [31]: this woman with HHT had an ischaemic stroke with hemiparesis in the third trimester of her pregnancy while she was being treated for PAVMs. The patient developed severe pulmonary insufficiency with post-partum haemothorax [31]. The complications were related to CAVMs and intracranial haemorrhage in 2 cases [13, 32], resulting in one foetal death.

Complications were related to hepatic AVMs (HAVMs) in 8 case reports [33–39]. There were 6 cases of acute decompensation heart failure due to hepatic involvement during pregnancy. In one case, a pregnant woman with previously unknown HHT was admitted for pre-term labour and cardiac failure. After a caesarean section, congestive signs improved with medical treatment [36]. In other cases of HHT undiagnosed during pregnancy, congestive heart failure improved spontaneously after delivery [34, 35, 37]. Three women underwent a post-partum liver transplant. In 1 case, the pregnant woman with known HHT had severe cholangitis and progressive liver dysfunction; delivery was induced in order to allow the liver transplant to be performed [40]. In 2 other cases, the women had hepatobiliary necrosis diagnosed on the basis of acute abdominal pain [38, 39]. One woman had digestive bleeding from a perforated gastric ulcer that could not be controlled endoscopically [14]. Branch retinal artery occlusion was observed in one case [41].

## Discussion

In the context of pregnancy, severe complications can occur in HHT women, and this literature review indicates that HHT pregnancies are likely to present significant maternal and/or foetal risks. One large study reported that the overall maternal death rate was 1.0% of pregnancies (95% confidence interval [0.1; 1.9]) [8] based on analysis of retrospective and prospective data and on data from the “relatives group”, defined as non-HHT women’s first degree relatives. Interestingly, all deaths occurred in women not under medical supervision for HHT. Furthermore, 2 case reports indicated a fatal maternal outcome in 2 cases [13, 14]. This maternal death rate is probably underestimated in cases of women undiagnosed before pregnancy. Severe maternal complications occurred in 2.7 to 6.8% of pregnancies [8, 10, 12] in the case series and 31 case reports published from 1986 to 2018. This indicates that, in women known to have HHT, and even if the majority of HHT pregnancies are uneventful, specific high-risk pregnancy monitoring is needed. Foetal death is very rare: among the case reports, 3 foetal deaths were reported [13, 16, 18], secondary to maternal complications, severe hypoxaemia with early-onset growth retardation at 25 weeks’ gestation in one case and maternal intracranial haemorrhage in the other case, with maternal death.

Pregnancy has a dramatic effect on the cardiovascular system and many complications in the HHT pregnancies occurred in the second and third trimesters at the time when peripheral resistance is reduced (by 35–40%), as is systemic vascular resistance [42]. Furthermore, given the physiological demands of utero-placental circulation and the developing foetus, there is an increase in cardiac output, with the biggest increase of up to 45% from baseline occurring during the first trimester. The increase in cardiac output slows late in the second trimester and drops slightly, late in the third trimester. We hypothesized that the complications in HHT patients during pregnancy related to pulmonary, liver and cerebral AVMs are favoured by these vascular changes in an abnormal vascular bed. Moreover, it has been shown by Rizvi et al. that, in patients with pulmonary AVMs - a chronic adapted state - evaluating arterial oxygenation can be falsely reassuring during pregnancy and haemoglobin is a vital determinant of arterial oxygen content that needs to be evaluated [43].

Screening and treatment of PAVMs before pregnancy is a priority because these are present in 25 to 50% of HHT women and can lead to haemothorax, which was the most frequent complication in the published case series and case reports. Incidence of haemothorax in 2 studies was 2.1% (4/185) (95% confidence interval 0.7–5.6%) [12], and 1.4% (6/484) (95% confidence interval 0.2–2.5%) [8] per pregnancy. However, as reported by Shovlin et al. [8], there was significant improvement in survival in women in whom the diagnosis of HHT or PAVMs had been made before pregnancy; all women in whom the diagnosis had been made previously, and who presented with haemothorax, survived. On the other hand, PAVM bleeds could be observed even in women who had been previously treated with embolotherapy before the pregnancy. Other complications related to PAVMs include haemoptysis, severe hypoxaemia, paradoxical emboli with brain abscess, paradoxical emboli with ischaemic cerebral stroke, paradoxical emboli with ocular ischaemia. Most complications from PAVMs occurred during the second or third trimester, which seems to be related to high cardiac output. In treated or untreated patients, specific clinical monitoring should be proposed, including regular measuring of oxygen saturation, and all patients should be informed of the risk of coughing up blood, which is not a nosebleed, and it should justify an immediate specialised consultation to exclude a PAVM rupture [8, 12].

Complications related to HAVMs were described in 8 case reports but were rare in case series. We can hypothesize that this difference is related to recruitment bias. The largest series published were from HHT expert centres specialised in pulmonology and internal medicine and not from hepato-gastroenterology or cardiology [8, 11, 12]. The most common complication of HAVMs in patients with HHT leads to high-output cardiac failure (6 cases [12, 34–37]), followed by hepatobiliary necrosis leading to liver transplant in 2 cases [38, 39]. Severe liver involvement in HHT is rare in young patients and usually clinically diagnosed through dyspnoea in women after the age of 50 years [44]. For this reason, complications are rare between 20 and 40 years of age even if cardiac output is higher during pregnancy, but easy to screen by liver Doppler ultrasound [45].

The risk of complications from cerebral or spinal AVMs was very low in the case series (4 intracranial haemorrhages [8, 12], no spinal haemorrhage) and case reports (2 intracranial haemorrhages [13, 32], no spinal haemorrhage). During pregnancy, spontaneous intracranial haemorrhage in all women occurs at a rate of 0.9 to 7.5 per 100,000 deliveries [46], which is higher than non-pregnant age-matched women. Even if CAVMs are frequent in asymptomatic HHT patients (15–25%) [47], it has been suggested that the risk of intracranial brain haemorrhage in HHT patients was lower than in sporadic CAVM patients [48]. This is not true for the risk of rebleeding, with a higher risk of re-rupture [49]. Screening for CAVMs is highly debated among HHT experts. The most recent HHT guidelines were drawn up in 2006 and concluded that there is no evidence for guiding the management of CAVMs during pregnancy and delivery [50]. The bleeding risk with CAVMs in HHT has been estimated retrospectively at 0.5% per year [48], but there are several cases of dramatic haemorrhage. Furthermore, even if the treatment can reduce risks, the prevention procedures have their own complication rate, and treatment techniques have treatment-associated morbidity and mortality [51, 52]. This was highlighted by the ARUBA trial (A Randomized Trial of Unruptured Brain Arteriovenous Malformations) that found that medical management alone was more effective than interventional therapy [51, 53]. We did not find any report of spinal AVM bleeding during pregnancy or more particularly at birth after epidural anaesthesia, even if it is a potential risk. Spinal AVMs are rare and have mostly been reported in children [54].

Finally, HHT can affect pregnancies with intra-uterine growth retardation and intra-uterine foetal death via chronic hypoxaemia, maternal hypovolaemia or maternal death. However, in the published series, miscarriage was observed in 14.4 to 20% during the first trimester [9, 11, 12], and prematurity in 9.4, 13.8 and 12% [9, 11, 12]. These figures were not significantly different from those of the control group, nor those found in the general population.

## Conclusion

Improving HHT diagnosis and information via reference centres and general information is an objective, and women should be educated about screening and possible pregnancy-related risks before becoming pregnant. In all cases, even if severe complications are rare, HHT pregnancies are high-risk and need reinforced monitoring from a maternal as well as a foetal point of view.

## Data Availability

The datasets used and/or analysed during the current study are available from the corresponding author on request.

## Abbreviations

ACVRL1 = ALK1: Activin receptor-like kinase 1
ARUBA: A Randomized trial of Unruptured Brain Arteriovenous malformations
AVM(s): Arteriovenous malformation(s)
CAVM: Cerebral arteriovenous malformation
ENG: Endoglin
HAVM: Hepatic arteriovenous malformation
HHT: Hereditary haemorrhagic telangiectasia
MADH4: Mothers against decapentaplegic homolog 4
PAVM: Pulmonary arteriovenous malformation
VEGF: Vascular endothelial growth factor
